# Mixed Education

**Published:** 1905-07-15

**Authors:** 


### MIXED EDUCATION.

Dr. Emil Reich, who has lately been delivering at the Hôtel Cecil an exceedingly interesting course of lectures on the Platonic philosophy, and on its applicability to the order of modern life, dwelt at length in one of these lectures upon the precepts laid down in the seventh book on laws, with reference to the education of children. Plato asserts that the sexes should be educated together up to the age of six years, and that, after that age has been reached, the education of boys should be entrusted only to men, and that of girls to women. Dr. Reich pointed out that in this, as in many other matters, the native sagacity of Plato worked under the guidance of experience; and he contrasted him, in this respect, with many modern writers and speakers, who have had little or no experience, and whose doctrines concerning education, like the German philosopher's description of a camel, have been evolved out of their moral consciousness. Ancient Greece was a small country, composed of many States differing from one another in laws and customs; and hence Plato had only to look around him in order to observe the effects which different principles of conduct actually produced. The education of the sexes together, for example, was carried to extreme lengths in Sparta, and the results were such as greatly to disturb the normal relations between them. Plato did not advocate that the education of girls should be such as to render them, in the objectionable sense, effeminate; and he even maintained that they should be able, in case of need, to wield weapons in defence of their country. What he did advocate was that the education of each sex should be in the hands of teachers of that sex as soon as the earliest period of childhood was past; and Dr. Reich criticised with considerable force the practical effects of the very different system pursued in the United States of America. There, as he told his audience, the education of both sexes, at least up to the age of sixteen or seventeen, is practically in the hands of women. The "schoolmarm" is an almost universal power in the land, and she teaches boys and young men, as well as girls and young women, and teaches them together. The result, according to the lecturer, is eminently unsatisfactory. The sort of romance or mystery which is the source of the most tender and the most abiding relations between the sexes never comes into existence; and the young men, while they learn to exercise the most exquisite politeness towards women, have little desire or relish for their society, and treat them with a sort of deference not unlike that which an untitled gentleman of old family may often be seen to extend to a peer of recent wealth and of new creation, with whom he has no special desire to cultivate acquaintanceship. The effect on both sexes is such as to leave a good deal to be desired, and it may perhaps serve to explain many of those peculiarities of the "Amurrican" girl which we in England think least worthy of imitation. In this country, however, according to the lecturer, we go somewhat into the opposite extreme; and he clearly would not approve of the unwritten law by which brothers and sisters, placed at different schools at Brighton or other health resort, are prohibited from recognising each other as they pass in the street. For the more wealthy classes the question is perhaps theoretical rather than practical; but it is one that may come into importance with the development of secondary education for the comparatively poor under the auspices of County Councils. The extent to which a teacher of either sex might be held to satisfy the needs of the more advanced pupils of both is an eminently controversial matter, and it is one very likely to be decided rather upon grounds of economy than upon grounds of principle. The principle to be borne in mind is clearly that the members of each sex should receive the best training for that fulfilment of their duties to the other which is also the fulfilment of their highest duties to the State; or, in other words, that young men and young women should retain the qualities which render them mutually complementary in mind and character, and fitted to be helpmates in the great work of training a future generation to be good mothers and good citizens. It is often said of a strenuous boy in Scotland that he will either make a spoon or spoil a horn; and it is probably true of many teachers that they have remarkable facilities for spoiling the raw material which is committed to them by parents or by the community. It clearly will not do to forget that, underlying the comparatively sexless "intelligent pupil" we have the future or undeveloped man or woman, and that our efforts should be directed to him or her as much as to the intelligence which may be one of his or her characteristics. A good deal of the ancient Greek philosophy has been so completely absorbed or assimilated by subsequent generations that we act upon it without knowing whence it is derived, and regard it as part of the common stock of human knowledge. Of education this is not quite true, and we shall assuredly do no harm by taking the experience and the teaching of Plato to our counsels.

